# Supplementary material for: Public Health Midwives’ perspectives on paediatric snakebite prevention and management in rural Sri Lanka: A qualitative study
Source: PLoS Negl Trop Dis. 2026 Feb 11;20(2):e0013976. doi: 10.1371/journal.pntd.0013976 (PMC12893596; doi:10.1371/journal.pntd.0013976)
Supplement: S1 File — (DOCX) [file pntd.0013976.s001.docx]

**Interview guide**

**Study title:** Public Health Midwives’ perspectives on paediatric snakebite prevention and management in rural Sri Lanka
**Study design:** Qualitative exploratory study using Focus Group Discussions (FGDs)

**Introduction for facilitator to read aloud**

Thank you for agreeing to take part in this discussion. We are here to learn about your experiences and views on preventing and managing snakebites in children in your community. There are no right or wrong answers — we want to hear about your real experiences, beliefs, and suggestions. Your responses will be kept confidential, and we will not record any names in our reports. The discussion will be audio-recorded for accuracy. You may choose not to answer any question or to stop the discussion at any time.

**Section A: Warm-up and background**

1. Please tell us about your role and daily work as a Public Health Midwife.
2. In your experience, how common are snakebites among children in your area?

**Probes**

- Recent cases you have heard about or been involved in.
- Seasonal or geographic patterns you have observed.

**Section B: Awareness and knowledge**

1. What do you know about the risks of snakebites in children compared to adults?
2. How do you usually identify a snakebite in a child?
3. Are you aware of the medically important venomous snakes in your area?
4. How did you acquire your knowledge on snakebites?

**Probes**

- Differences in signs and symptoms between children and adults.
- Understanding of venom types and urgency of treatment.

**Section C: Community beliefs and practices**

1. What beliefs or traditions about snakebites are common in your community?
2. How do these beliefs influence how parents respond when a child is bitten?
3. Have you observed any traditional or home-based first aid being used?

**Probes**

- Role of elders, religious leaders, or traditional healers.
- Examples of myths or spiritual beliefs about snakes.
- Positive or negative outcomes from traditional practices.

**Section D: First-aid knowledge and skills**

1. If a child is bitten by a snake, what steps would you advise or take?
2. Have you received any training on first aid for paediatric snakebites?
3. How confident do you feel in teaching or applying first aid in such cases?

**Probes**

- Correct sequence of actions (immobilization, reassurance, transport).
- Awareness of pressure immobilization techniques.
- Fear or hesitation in providing first aid.

**Section E: Role perception and challenges**

1. What do you see as your role in preventing snakebites in children?
2. What challenges do you face in educating families about snakebite prevention?
3. Are there any environmental or systemic issues that make your work harder?

**Probes**

- Workload and competing duties.
- Language or communication barriers.
- Transport difficulties (e.g., bad roads, floods, elephants).

**Section F: Systemic and policy-level factors**

1. What facilities or services are available for treating children with snakebites locally?
2. Are there any gaps in the health system that affect timely treatment?
3. What additional support would help you in your role?

**Probes**

- Training opportunities and resources.
- Ambulance and referral systems.
- Coordination with hospitals and other health staff.

**Section G: Recommendations**

1. What do you think should be done to reduce snakebites in children?
2. How can communities be better educated about prevention and first-aid?
3. What role should schools, religious institutions, or community groups play?

**Closing questions**

1. Is there anything else you would like to share about your experiences with paediatric snakebite prevention or management?
2. Do you have suggestions for how these findings should be shared with health authorities or communities?
