# Supplementary material for: Public Health Midwives’ perspectives on paediatric snakebite prevention and management in rural Sri Lanka: A qualitative study
Source: PLoS Negl Trop Dis. 2026 Feb 11;20(2):e0013976. doi: 10.1371/journal.pntd.0013976 (PMC12893596; doi:10.1371/journal.pntd.0013976)
Supplement: Attachment — Submitted filename: Response to reviewers.docx [file pntd.0013976.s003.docx]

**Authors’ response to reviewers’’ comments**

Dear editor and the reviewers,

We would like to extend our heartfelt thanks to the reviewers for their time, effort, and invaluable feedback in reviewing this manuscript. Your insightful comments and constructive suggestions have significantly enhanced the quality of this work. We greatly appreciate your expertise and dedication, which have contributed to refining our study. Your support in the review process is deeply appreciated, and we are grateful for your commitment to advancing research in this important area.

Kavinda Dayasiri

Corresponding author

Please see the responses below for specific comments.

Journal Requirements:

1) Please ensure that the CRediT author contributions listed for every co-author are completed accurately and in full.

At this stage, the following Authors/Authors require contributions: Kavinda Chandimal Dayasiri. Please ensure that the full contributions of each author are acknowledged in the "Add/Edit/Remove Authors" section of our submission form.

The list of CRediT author contributions may be found here: <https://journals.plos.org/plosntds/s/authorship#loc-author-contributions>

Thank you, we have updated the name of the respective authors and their contribtions.

2) Please upload all main figures as separate Figure files in .tif or .eps format. For more information about how to convert and format your figure files please see our guidelines:

<https://journals.plos.org/plosntds/s/figures>

Thank you and done.

3) Some material included in your submission may be copyrighted. According to PLOSu2019s copyright policy, authors who use figures or other material (e.g., graphics, clipart, maps) from another author or copyright holder must demonstrate or obtain permission to publish this material under the Creative Commons Attribution 4.0 International (CC BY 4.0) License used by PLOS journals. Please closely review the details of PLOSu2019s copyright requirements here: PLOS Licenses and Copyright. If you need to request permissions from a copyright holder, you may use PLOS's Copyright Content Permission form.

Please respond directly to this email and provide any known details concerning your material's license terms and permissions required for reuse, even if you have not yet obtained copyright permissions or are unsure of your material's copyright compatibility. Once you have responded and addressed all other outstanding technical requirements, you may resubmit your manuscript within Editorial Manager.

Potential Copyright Issues:

- Figure 1. Please (a) provide a direct link to the base layer of the map (i.e., the country or region border shape) and ensure this is also included in the figure legend; and (b) provide a link to the terms of use / license information for the base layer image or shapefile. We cannot publish proprietary or copyrighted maps (e.g. Google Maps, Mapquest) and the terms of use for your map base layer must be compatible with our CC BY 4.0 license.

Note: if you created the map in a software program like R or ArcGIS, please locate and indicate the source of the basemap shapefile onto which data has been plotted.

If your map was obtained from a copyrighted source please amend the figure so that the base map used is from an openly available source. Alternatively, please provide explicit written permission from the copyright holder granting you the right to publish the material under our CC BY 4.0 license.

If you are unsure whether you can use a map or not, please do reach out and we will be able to help you. The following websites are good examples of where you can source open access or public domain maps:

* U.S. Geological Survey (USGS) - All maps are in the public domain. (http://www.usgs.gov)

* PlaniGlobe - All maps are published under a Creative Commons license so please cite u201cPlaniGlobe, http://www.planiglobe.com, CC BY 2.0u201d in the image credit after the caption. (http://www.planiglobe.com/?lang=enl)

* Natural Earth - All maps are public domain. (http://www.naturalearthdata.com/about/terms-of-use/).

Thank you very much!! We replaced Figure 1 and renamed it as follows

“Figure 1. Distribution of the study settings in Sri Lanka. Map base layer from the U.S. Geological Survey (Public Domain), available at: https://earthexplorer.usgs.gov/”

Reviewers' Comments:

Reviewer's Responses to Questions

Key Review Criteria Required for Acceptance?

As you describe the new analyses required for acceptance, please consider the following:

Methods

-Are the objectives of the study clearly articulated with a clear testable hypothesis stated?

-Is the study design appropriate to address the stated objectives?

-Is the population clearly described and appropriate for the hypothesis being tested?

-Is the sample size sufficient to ensure adequate power to address the hypothesis being tested?

-Were correct statistical analysis used to support conclusions?

-Are there concerns about ethical or regulatory requirements being met?

Reviewer #1: Nil issues

Thank you very much for the comment!!

Reviewer #2: The study’s objectives are clearly stated and well aligned with the research focus. Focus group discussions are appropriate for exploring community health workers’ perspectives. The population and data collection are described in detail.

The methodology demonstrates strong rigor, with effective use of reflexivity, triangulation, and member-checking. Analysis is clearly presented, supported by a code tree and evidence of thematic saturation. Ethical clearance is properly obtained; no ethical concerns identified.

Thank you very much for the comment!!!

This study appears to be part of broader research on pediatric snakebite management in rural Sri Lanka including physician perspectives, parental knowledge, and preventive practices. Briefly stating this context would help situate the current study and highlight its contribution within a comprehensive research effort.

Thank you very much for the comment!! We added the following paragraph (paragraph 3) in the introduction section with the updated references 6-10 in order to present the context to the current study.

“Building on earlier components of this research programme—which highlighted poor parental knowledge of snakebite first aid, reliance on traditional healing practices, and delays in seeking formal care for affected children in rural Sri Lanka —this study turns attention to a critical but understudied cadre within the public health system: Public Health Midwives. While previous findings also revealed gaps in clinical preparedness and systemic challenges in responding to paediatric envenoming, little is known about how PHMs perceive their role in prevention or early response, particularly beyond their formal mandate of supporting maternal and child health services for children under five. Given their close engagement with families and established trust within communities, PHMs may represent a valuable yet underutilized resource in reducing paediatric snakebite harm. This study explores their knowledge, experiences, and perceived barriers in addressing paediatric snakebites, and captures their recommendations for improving prevention and early care strategies within their operational realities.”

Reviewer #3: The objective of this study was to explore the insights of Public Health Midwives (CHWs) into pediatric snakebite prevention and management in 2 high-incidence districts in Sri Lanka, focus group interviews with qualitative analysis was an appropriate design for this state objective. The authors clearly describe the study participants and with 11 focus groups and a total of 74 participants this is an impressive sample particularly for qualitative analysis. Authors clearly outlined their process for meeting with ethical requirements.

Thank you very much for the comment!!!

Reviewer #4: Yes

Reviewer #5: Important components are missing in the methodology followed, see questions below

Thank you very much, we have gone through the comments and responded to all comments with updated revisions.

Results

-Does the analysis presented match the analysis plan?

-Are the results clearly and completely presented?

-Are the figures (Tables, Images) of sufficient quality for clarity?

Reviewer #1: This is very long and the readership of this journal is going to struggle to read thru, and in fact may only read No 1 and miss much of the important material. This should be shortened (maybe to a 1/3 of the length), and the remaining material needs to be in a Supplement. This is not a qualitative research journal.

Thank you very much for the comment. We have done major reductions in the contents in both Results (including omission of the second-order analysis) and Discussion section. Key results (major themes) were left within the manuscript following the recommended revisions.

Reviewer #2: The results are clearly and well presented, highlighting key barriers such as knowledge gaps, limited experience, and structural barriers. The recommendation for pediatric-specific training and broader system improvements is well justified. A second-order analysis has been conducted, adding depth to the findings.

Thank you very much for the comment!!

Figure 1 – This same figure appears to have been previously published in:

Dayasiri, K., Perera, T., Gawarammana, I., & Jayamanne, S. (2025). Caught between fear and tradition: parental knowledge, beliefs and emergency responses to paediatric snakebites in rural Sri Lanka. BMJ Paediatrics Open, 9(1), e003658. https://doi.org/10.1136/bmjpo-2025-003658.

Please clarify whether this is a reused figure and, if so, ensure proper attribution and confirm that permission has been obtained for reuse in the current manuscript.

Thank you very much for the comment!! We have now replaced Figure 1 with newly created map showing the study settings.

Reviewer #3: Thematic analysis is an appropriate strategy for this qualitative study; but I do think the authors could be a bit more creative and effective in methods of presentation and visualization of qualitative data (eg thematic maps or network). The results were presented in a coherent and organized fashion and the use of direct quotes was highly effective.

Thank you very much for the comment. We have replaced the current version of the coding tree with a thematic map.

Reviewer #4: Yes, but see the comments

Reviewer #5: No, authors discuss data in the results which combined with the methodology makes it even harder to assume information from FGD was really participant driven. And these discussions are then repeated in discussion section.

Thank you very much for the comment!! Our results section is grounded entirely in participant-driven data, derived from verbatim transcripts of 11 focus group discussions (FGDs) conducted with 74 Public Health Midwives (PHMs) across two high-incidence snakebite districts. Each theme and subtheme in the results is substantiated with direct quotations from participants. To protect participant confidentiality, we have intentionally omitted all identifiable details in the manuscript, as per our ethical approval conditions (ERC/PGIM/2024/080). The raw data, including transcripts with identifiable information, have been securely stored and are available for audit by the journal under appropriate ethical safeguards if required.

We agree there are repetitions in discussion and have therefore, revised the results and discussion sections to avoid repetition.

Conclusions

-Are the conclusions supported by the data presented?

-Are the limitations of analysis clearly described?

-Do the authors discuss how these data can be helpful to advance our understanding of the topic under study?

-Is public health relevance addressed?

Reviewer #1: Good

Thank you very much for the comment!!

Reviewer #2: The conclusions are supported by the data presented. However, the section could be strengthened by more explicitly outlining how the suggested improvements—such as pediatric-specific training and system-level changes—might be implemented in practice, as partly discussed in the results and discussion sections.

Thank you for the comment!! We have rewritten the conclusion as mentioned below “Public Health Midwives (PHMs) in rural Sri Lanka are well-positioned to support paediatric snakebite prevention but face structural barriers, inadequate training, and competing responsibilities. Addressing these challenges requires more than additional duties—it demands system-level support. This includes paediatric-specific, hands-on training; integration of snakebite education into routine child health services; culturally appropriate materials; and improved referral systems. Given PHMs’ willingness to act, immediate next steps should include developing and piloting a community-based intervention package—combining training, decision-support tools, and structured outreach—in collaboration with public health authorities. Such efforts must be embedded within existing workflows to avoid overburdening an already stretched workforce. Strengthening primary care systems alongside PHM capacity can ensure sustainability, relevance, and community impact. While this study focused only on PHMs, our findings highlight an actionable pathway to reduce paediatric snakebite morbidity and mortality—through targeted investments in the frontline workforce and practical, scalable models of care suited to rural health systems.”

The limitations are appropriately acknowledged. The authors clearly demonstrate how the findings contribute to advancing our understanding of pediatric snakebite management from the perspective of healthcare workers. The study has clear public health relevance, particularly for improving care in rural, high-risk settings within the global snakebite belt.

Thank you very much for the comment!!

Reviewer #3: Discussion and conclusions are well-summarized and consistent with data presented in the study, although I do think they should strengthen the emphasis in their conclusion regarding providing adequate structural supports and addressing the challenges with limited health workforce and competing priorities that were highlighted in the results and are a well-documented challenge with continue to layer on additional programs and responsibilities on frontline polyvalent CHWs. Authors highlighted limitations in terms of only including PHMs in the focus groups. I would love to see the authors strengthen the conclusions with immediate next steps based on these study findings (ie developing and piloting a pediatric snake bite intervention focused on equipping PHMs) given the public health relevance.

Thank you for being spot on. Yes, currently we are conducting a quasi-experimental trial on the effectiveness of a brief educational intervention delivered to Public Health Midwives in improving the snakebite prevention practices, first-aid practices and pre-hospital management among the parents they serve. The study is currently underway!!

We have rewritten the conclusion as mentioned below “Public Health Midwives (PHMs) in rural Sri Lanka are well-positioned to support paediatric snakebite prevention but face structural barriers, inadequate training, and competing responsibilities. Addressing these challenges requires more than additional duties—it demands system-level support. This includes paediatric-specific, hands-on training; integration of snakebite education into routine child health services; culturally appropriate materials; and improved referral systems. Given PHMs’ willingness to act, immediate next steps should include developing and piloting a community-based intervention package—combining training, decision-support tools, and structured outreach—in collaboration with public health authorities. Such efforts must be embedded within existing workflows to avoid overburdening an already stretched workforce. Strengthening primary care systems alongside PHM capacity can ensure sustainability, relevance, and community impact. While this study focused only on PHMs, our findings highlight an actionable pathway to reduce paediatric snakebite morbidity and mortality—through targeted investments in the frontline workforce and practical, scalable models of care suited to rural health systems.”

Reviewer #4: See comments

Reviewer #5: No, the midwives indicate being overburdened and needed extra staff to be able to pick up snakebite education and implementation. They also suggest other community platforms instead. Yet the researchers state the midwives to be an underutilised workforce and suggest global involvement of this workforce without further context.

Thank you for the comment. We agree that one may disagree with the conclusion in the current format as the conclusion is not clearly written with a clear message. Therefore, we have rewritten the conclusion section to make the message clearer. Please see the revised conclusion.

Editorial and Data Presentation Modifications?

Use this section for editorial suggestions as well as relatively minor modifications of existing data that would enhance clarity. If the only modifications needed are minor and/or editorial, you may wish to recommend “Minor Revision” or “Accept”.

Reviewer #1: Abbreviations: avoid these, particularly things like FGD because qualitative research will not be familiar to many readers and it should be written in full.

Thank you very much!! We have revised and written the expanded version ‘focused group discussion’ instead of FGD throughout the manuscript.

Please use the terms snake and snakebite correctly. This is an unfortunately use of English that is creeping into the snake literature. There is no such thing as “Snakebite envenoming” – it should be “snake envenoming” or snakebite that causes envenoming. And the term “snakebite endemic” is even worse. Describe what you mean – endemic means that it comes from that area – of course this is the case with snakebite like this, unless the authors are distinguishing this from exotic snakebite. Any district will have “endemic” snakebites. I assume the authors simply mean that there is a high rate of snakebites in these regions – just say that.

Thank you very much!! These terms are now correctly mentioned throughout the manuscript.

Abstract

The conclusion is long and needs to be shortened with the key points, and not simply restating the Results.

Thank you very much!! The conclusion section is rewritten as per the reviewers’ comments.

Reviewer #2: (No Response)

Reviewer #3: It would be helpful to add some more concrete epidemiologic statistics to the introduction on the burden (ie incidence, morbidity, mortality) of pediatric snake bites in Sri Lanka or the region.

Thank you. We added the following information in introduction section to present more concrete epidemiological data regarding snakebites in Sri Lanka. The information is supported by reference 4.

“A nationwide community‐based survey in Sri Lanka estimated the crude annual incidence of snakebite at 398 per 100 000 population, with envenoming occurring in 151 per 100 000 and a mortality rate of 2.3 per 100 000 . The study extrapolated these figures to suggest approximately 80500 snakebites, 30500 envenomings, and about 460 deaths annually across the country. Spatial risk mapping further revealed significant geographic variation underscoring the need for region‑specific prevention and treatment strategies.”

Unfortunately, we do not have national level concrete data regarding paediatric snakebites. This is supported by reference 3 that presents paediatric epidemiological data.

I would suggest the authors include the range of number of participants/focus group and in table 1 also include native language of the CHWs. I could not view the supplemental coding tree document.

Native language of the PHMs is given in Table 1. Thematic map is included as a supplementary material. Please see the supplementary material.

Please mention how many participants/focus group.

Thank you for the comment!! We conducted 11 focussed group discussions and recruited 74 Community health Workers to the study. This is mentioned in the ‘Methods’ section.

Please clarify if the same investigators who facilitated the FGDs were also involved in the coding/thematic analysis.

Thank you, yes it was the same investigators. The following section was added in data analysis section to describe data analysis clearly.

“Two investigators who facilitated the FGDs also conducted the initial coding and thematic analysis. To enhance rigor and minimize bias, coding was performed independently by both researchers, with discrepancies resolved through discussion.”

Reviewer #4: General comments

This study denotes a very important scientific exercise. The topic is very important, urgent and highly neglected. A much-needed exploration with regard to the public health and paediatrics in the context and beyond. As a qualitative exploration, the study reveals valuable and vast knowledge of a broad scope admitting the limitations. In general, the manuscript is well written except for the introduction component. I would suggest the authors to reveal more the subthemes of the analysis as described below. Detail comments follow.

Thank you very much for the comment. We have responded to your specific comments below.

Comment No. LN Comment

01 Abstract The statement, “Public Health Midwives (PHMs) serve as trusted community health workers and are uniquely positioned to support prevention and early response to paediatric snakebites”: It is quite true that the PHMs are trusted community health workers. However, whether they are uniquely positioned to support and prevent paediatric snake bite should be a finding of this study. The PHM’s serve on routine maternal and child health activities and they have set areas to cover within their services. There have been lot of instances where, PHM’s are overloaded with extra work and they follow these with many constraints. So stating that they are uniquely positioned for this need to be carefully sorted within the research.

Thank you for the suggestion – we have revised the relevant section in the abstract as “Snake envenoming remains a significant yet under-addressed public health issue in rural Sri Lanka, where children face disproportionately severe outcomes. Public Health Midwives (PHMs) serve as trusted community health workers. While they may be well placed to support paediatric snakebite prevention and early response, their actual capacity and role in this regard remain underexplored.”

02 Abstract Results section can be more improved by expressing one major finding under each theme (you may not elaborate the wordy themes here but can mention briefly the six themes) than just listing the major themes. This could be challenging under the word count, but would upgrade the study much more as the audience could have a glimpse of what the study really reflect.

Thank you very much – we have revised the results section as you suggested and within the word count limits as “Thematic analysis revealed five major themes. PHMs reported limited awareness of paediatric-specific snakebite risks and described frequent community reliance on traditional beliefs that delayed care. Their first-aid knowledge was often outdated or incomplete, and they lacked practical training. Operational challenges—such as excessive workload, language barriers, and poor infrastructure—limited outreach. Despite these constraints, PHMs expressed strong motivation to engage in prevention through schools and home visits. They proposed feasible solutions including targeted training, improved transport and referral systems, and culturally appropriate health education materials, highlighting their readiness to contribute if supported with system-level interventions.”

03 Abstract Could you please make the conclusion concise and focused?

Thank you and we have revised the conclusion in abstract as mentioned below

“Public Health Midwives in rural Sri Lanka have strong potential to reduce paediatric snakebite harm but face key systemic barriers. Effective prevention requires targeted training, supportive tools, and integration into existing services without adding workload. Strengthening PHM capacity within primary care systems offers a practical, sustainable approach to improve outcomes in these communities.”

04 Author summary LN 62 The abstract mentions five major themes while here authors state six. Please correct.

Thank you, we have corrected it as five.

Introduction

05 Introduction The introduction of this manuscript is very thin. Ideally the introduction should start broad and consist of stating the problem, implications of the problem and describing the problem in the local context, properly stating the research gap and narrowing down to justifying the study. Within this descriptions it would be valuable if the authors define what age range “paediatric” refers to, describe the epidemiology of paediatric snakebite globally and locally and describe the implications including if any, different manifestations from adults, different manangement and different burden reported briefly.

Thank you very much for the suggestion. Please see the revised section – first paragraph in Introduction as mentioned below supported references 1-3.

“Snakebite envenoming is a neglected tropical disease that disproportionately affects rural and impoverished populations across the globe, causing over 5 million bites and up to 138,000 deaths annually, with many more left with permanent disabilities. Children, typically defined as individuals aged 0–18 years, are particularly vulnerable due to their smaller body mass, greater outdoor exposure, and limited ability to respond to danger. Paediatric snakebites account for a substantial proportion of cases in endemic regions, with higher risks of severe envenoming, long-term disability, and death compared to adults. Despite these risks, paediatric-specific data on snakebite incidence, outcomes, and prevention strategies remain limited in most settings.”

06 Introduction LN84-90 I would suggest the authors to be more transparent with the scope of work of PHMs. The PHMs are the grassroot level maternal and child health care workers serving eligible families in a community. This will mean that they will cater the families with pregnant women and children aged upto five years. Disease surveillance is not a direct duty applied to PHMs but for Public Health Inspectors. So, whether the PHM’s are motivated, willing and capable to work for snakebite prevention and management should be a finding of the study and not the authors opinion at the beginning. Authors can highlight this as a research gap further. Please be precise and appropriate with regard to this paragraph.

Thank you and we have updated the relevant section in introduction as mentioned below

“Public Health Midwives (PHMs) are frontline health workers in Sri Lanka’s public health system, primarily responsible for providing maternal and child health services to pregnant women and children under five years within their communities. While disease surveillance is typically the role of Public Health Inspectors, PHMs’ close contact with families positions them as potential agents for health education and early intervention. However, their preparedness, motivation, and capacity to engage in paediatric snakebite prevention and management have not been thoroughly explored.”

07 LN 110-11 Can the authors justify why they selected only PHMs of the public health officers? Why not PHIs? Specifically relating to their active role in school health promotion given the age range of children covered? Is it for feasibility?

Thank you for the comment, the following section was added in Study Design to justify the inclusion of PHMs “PHMs were selected given their unique household-level access to families, especially those with young children, and their continuous engagement in maternal and child health services within the community.”

08 LN 123-134 Authors can reduce repetition on purposive sampling a little further.

Thank you. The two respective paragraphs were revised to avoid repetition.

09 LN 133 Will it be more appropriate to mention 5-7 participants per focus group amounting to 11 FGDs with a total of 74 PHMs?

Thank you. We revised this as advised “A total of 74 PHMs were purposively selected to participate in eleven focus group discussions (FGDs), with each group comprising 5 to 7 participants.”

10 LN172 Authors claim “Triangulation of data sources”. The data source in the study is the FGDs of PHMs. Is the above statement valid as there are no multiple data sources in the study?

Thank you for the comment. Although the study collected data solely through focus group discussions with PHMs, triangulation was achieved through multiple analysts independently reviewing and interpreting the data to enhance credibility.

We revised the relevant section accordingly “Reflexivity, triangulation of analysts, and use of direct quotations helped enhance analytic trustworthiness.”

Results

11 LN 176 It is really good to describe the participants. As this is a qualitative manuscript, I would recommend to briefly include the personal characteristics of participants in a paragraph text rather than a table. But author’s/Editors can decide on this. How many PHMs took care of Sinhala and other ethnic communities respectively?

Thank you, we presented data in Table 1 as a single paragraph.

“A total of 74 Public Health Midwives participated in the focus group discussions. The majority were between 30 and 39 years old (39.2%, n = 29), followed by those aged 40–49 years (36.5%, n = 27), 20–29 years (10.8%, n = 8), and 50–59 years (13.5%, n = 10). In terms of work experience, 32.4% (n = 24) had less than five years of service, 28.4% (n = 21) had between 5 and 10 years, 24.3% (n = 18) had between 10 and 15 years, and 14.9% (n = 11) had more than 15 years of experience. Less than half of the participants (41.9%, n = 31) reported having previously provided snakebite-related health education to parents. Only a small number (6.8%, n = 5) had received prior training specifically on first aid for paediatric snakebites. Participants were drawn from two high-risk districts: 47.3% (n = 35) from Polonnaruwa and 52.7% (n = 39) from Ampara.”

Although over approximately 25 PHMs served both Sinhala and Tamil-speaking communities, we did not collect detailed data on the ethnic composition of the populations each PHM served.

12 LN179 Is it an inductive thematic analysis? Or a deductive according the pre-defined themes? It will be good to mention this.

Thank you very much. It was an inductive thematic analysis. We mentioned it within the same sentence.

13 LN 180 -192 The authors state the specific risks for paediatric snake bite. Please transfer this part to the introduction and elaborate it more within the introduction as this is the results section. You can also discuss this in the discussion.

Thank you, we removed these paragraphs from results section. Due to concerns raised by other reviewers regarding the word count of the manuscript, we did not expand the introduction and discussion sections any further.

14 LN 224-226 Move to discussion

Thank you, we have moved this to Discussion – please see paragraph 4 in Discussion

15 LN 271-273 It is of course a challenge in qualitative research presentations to demarcate between the results and the discussion. The statement included “These findings highlight the complex interplay between tradition, familial hierarchy, and healthcare behaviour, suggesting that any intervention aiming to improve paediatric snakebite outcomes must be culturally sensitive and developed with local context in mind. Simply dismissing these beliefs risks alienating communities”, would be better stated as an opinion of the PHM’s rather than the researcher’s in the results section, if that is the case. Otherwise move to the discussion.

Thank you, we revised the paragraph as follows “PHMs described how traditional beliefs, family hierarchies, and community norms strongly influenced healthcare-seeking behaviours following paediatric snakebites. They felt that efforts to improve outcomes must be rooted in cultural understanding and shaped by the local context. According to the PHMs, dismissing these beliefs outright could alienate families and reduce the effectiveness of interventions. Instead, they stressed the importance of introducing counter-narratives that are respectful of tradition while promoting evidence-based care, ideally through collaboration with trusted community leaders.”

16 LN 389 Elaborate the abbreviation PHI

Thank you, it is now elaborated as Public Health Inspectors

17 LN 407 onwards (results section) The second order analysis: This might be a good attempt, but adds lot of repetition and jargon in the results section (reflecting doubt of over using language models). I would rather incorporate the statements here within the same results section. One reason is anyway the authors reflect the patterns within the previous sections. If the authors believe that this section should be preserved, mention about this in the methods as well. With the relatively long results section, I would suggest either to incorporate this component while presenting the former results or focus on patterns and provide a concise paragraph for the section avoiding repetition. Did the authors find patterns between the PHMs reflections within different community groups/ cultures or geographies? Would be interesting to highlight this.

Thank you very much for the comment. We decided to remove the section on second-order analysis due to risk of repetition, use of jargon and increased length of the results section. This was also suggested by other reviewers.

18 Results (General) The authors describe the results under the major themes. Although we can clearly identify the subthemes within each major theme, these are not separately stated. The current version of presenting the results section is reader friendly than incorporating specific subthemes within the text. However, if the authors can include a figure to showcase the subthemes reflecting different facets under each major theme, the readers would be able to visualize the findings of the study very directly and easily rather than reading the whole article. One of the aims of thematic analysis is to present this clarity and diversity among the participant opinions to the audience. While having a rich data set, It would be valuable to include a thematic figure representing the subthemes.

Thank you very much. We have now provided a thematic map as a supplementary material.

19 Results (General) Did the researchers find any direct objections/ barriers or negative attitudes reported by the PHMs on incorporating snakebite prevention to their list of duties? Would be interesting to know.

Thank you for this insightful query. No direct objections or negative attitudes were expressed by PHMs regarding the incorporation of snakebite prevention into their existing duties.

Discussion

20 First paragraph Ideally the first paragraph of a results section should reflect the significance of the study. Rather than highlighting that PHM’s are ready to work for snakebite prevention, which needs to be interpreted more carefully with a broader study involving the health system, this study is unique as the interviews provide us with a broad tapestry of knowledge ranging from personal beleifs, opinions, practical problems, culture and context to policy level barriers on prevention of paediatric snakebite. I guess this is one of the very few qualitative explorations on field level experiences on the topic. Authors can ofcourse state that PHM’s exert responsibility and enthusiasm on providing services. It would be valuable to reflect this depth and gravity in the introductory paragraph of the discussion.

Thank you very much. We replaced the first paragraph in Discussion to highlight what the reviewer has suggested. The first paragraph is as follows. “This study provides one of the few qualitative explorations into frontline, field-level experiences of paediatric snakebite prevention and management in Sri Lanka, offering a rare and nuanced view that spans personal beliefs, practical realities, cultural influences, and policy-level barriers. Through in-depth focus group discussions with Public Health Midwives (PHMs) working in two high-incidence districts, we captured a broad tapestry of perspectives that illuminate how snakebite risk, prevention, and care are understood and navigated within rural communities. The findings reveal not only the operational challenges and systemic gaps that limit effective response, but also the embedded cultural beliefs and social dynamics that shape care-seeking behaviour. While PHMs demonstrated a strong sense of responsibility and enthusiasm for engaging in prevention, their experiences underscore the need for contextually grounded, health system–integrated interventions that address the interplay between biomedical guidance, cultural traditions, and structural constraints.”

Conclusions

21 LN500-501 I would like the authors to revise the statement “PHMs in rural Sri Lanka are uniquely positioned to lead community-level paediatric snakebite prevention and response” which as I mentioned earlier should be a conclusion of a much broader exploration. But the authors can conclude their willingness, enthusiasm and perceived responsibility, highlighting the pragmatic gaps and valuable policy implications elaborated by them.

Thank you very much. We have revised the conclusion as follows “PHMs in rural Sri Lanka expressed clear willingness, enthusiasm, and a strong sense of responsibility toward engaging in paediatric snakebite prevention and response. However, their accounts also revealed significant pragmatic gaps—most notably in paediatric-specific knowledge, practical training, and operational resources—that constrain their ability to translate this commitment into consistent practice. Importantly, their insights offered valuable, contextually grounded recommendations with direct policy relevance, including integrating snakebite education into existing maternal–child health activities, strengthening school- and community-based awareness, and addressing systemic barriers to timely care. Given PHMs’ willingness to act, immediate next steps should include developing and piloting a community-based intervention package—combining training, decision-support tools, and structured outreach—in collaboration with public health authorities. Such efforts must be embedded within existing workflows to avoid overburdening an already stretched workforce. Strengthening primary care systems alongside PHM capacity can ensure sustainability, relevance, and community impact. While this study focused only on PHMs, our findings highlight an actionable pathway to reduce paediatric snakebite morbidity and mortality—through targeted investments in the frontline workforce and practical, scalable models of care suited to rural health systems.”

22 Discussion (general) Could the authors add a separate paragraph on country level policy gaps identified by the PHMs? Or organize the facts into one para?

Thank you very much. Please see paragraph 2 in revised Discussion.

23 LN505 Change “offer a promising path” to “may offer a promising path” as such conclusions cannot be driven directly from exploratory qualitative studies.

Thank you very much. We have now entirely rewritten the conclusion section as also suggested by other reviewers.

Other

24 Title The title phrase, “Bridging tradition and evidence” Does not seem to shed light directly on what the study denotes. It also reflect a too similar pattern with the already available publications on different components of the topic.

The title is revised as “Public Health Midwives’ perspectives on paediatric snakebite prevention and management in rural Sri Lanka: a qualitative study”.

The short running title is also revised accordingly.

25 PPIE Could the authors mention about Patient Public Involvement in this study

The following statement was included in Methods section “Patients and/or the public were not involved in the design, or conduct, or reporting, or dissemination plans of this research.”

Reviewer #5: (No Response)

Summary and General Comments

Use this section to provide overall comments, discuss strengths/weaknesses of the study, novelty, significance, general execution and scholarship. You may also include additional comments for the author, including concerns about dual publication, research ethics, or publication ethics. If requesting major revision, please articulate the new experiments that are needed.

Reviewer #1: Nil further

Reviewer #2: Overall, the study provides valuable insights into community-level pediatric snakebite prevention and response, shedding light on both structural and cultural barriers that influence care in high-risk rural settings. However, since this study appears to be part of broader research on pediatric snakebite management in rural Sri Lanka—including physician perspectives, parental knowledge, and preventive practices—briefly stating this context would strengthen the manuscript.

Thank you very much. We have described this context in Introduction section with references to support it. Please see last paragraph in Introduction section.

Specific comments:

Line 230 – The abbreviation “PHM” has already been introduced multiple times (e.g., Lines 28, 58, 85, 111, 187, 299, and 349). Consider avoiding repeated redefinitions unless necessary for clarity.

Thank you very much. We have corrected this throughout manuscript.

Line 389 – The abbreviation “PHIs” is unclear. Please clarify its meaning at first use—does it refer to Public Health Inspectors?

Thank you very much for the comment. We have expended it as ‘Public Health Inspectors’ in the same line.

Reviewer #3: This is a well-designed and well-written study on the perspectives of CHWs on pediatric snake-bite prevention and management in a high-incidence region of Sri Lanka. While the methods were not particularly novel, it was a very well-conducted and clearly described and I think this community-grounded approach was highly effective in better understanding the nuances of pediatric snake bite perspectives as well as identify potential opportunities for intervention.

Thank you very much for the comment.

Reviewer #4: General comments

This study denotes a very important scientific exercise. The topic is very important, urgent and highly neglected. A much-needed exploration with regard to the public health and paediatrics in the context and beyond. As a qualitative exploration, the study reveals valuable and vast knowledge of a broad scope admitting the limitations. In general, the manuscript is well written except for the introduction component. I would suggest the authors to reveal more the subthemes of the analysis as described below. Detail comments follow

Thank you very much, we have entirely re-written the introduction section as suggested by most reviewers. A thematic map is annexed as supplementary material.

Reviewer #5: Study looking at the role of public health midwives as trusted community health workers and their potential in supporting snakebite prevention and response.

Introduction: the context is missing; on snakebite incidence in children and on health systems in communities; what’s the role of the midwife and how does this compare to other health care providers in the community setting. We don’t know why the authors decided to focus on the midwives.

Thank you for the comment. We have described both global and Sri Lankan context regarding paediatric snakebites in the Introduction section. Due to increased word counts (as pointed out by reviewers), we were careful not to write lengthy paragraphs regarding them. We have justified inclusion of PHMs both in the Introduction and the study design section in Methods.

Methods: Please use a checklist such as COREQ to ensure adequate reporting of this qualitative report. This will help identifying missing information, such as

- What was the methodological orientation stated to underpin the study eg grounded theory, discourse analysis, ethnography, phenomenology, content analysis

- How were participants selected, how many refused to participate etc

Thank you very much, a COREQ checklist is now provided as supplementary material

Interview: the interview was added but there’s no info on prompts used and only few questions are listed. Without the interview guide and prompts it’s difficult to see how researchers ensured information collected was coming from the participants instead of being influenced/steered in a direction by the interviewers.

Thank you very much. Interview guide is now attached as supplementary material.

There’s no information on data saturation.

Thank you. Data saturation was achieved after the ninth FGD. This is reported under Data Analysis section in Methods.

Results:

Table 1 please present data as continuous variables instead of in categories, eg mean age with SD instead of grouping.

The mean age of participants was 39.8 years (SD = 8.6). This is now reported in a paragraph under participant characteristics in Results section.

There’s a lot of overlap in the results and discussion. A substantial part of the results section is actually a discussion (eg 271-276 or 344-348). These then get repeated in the discussion section.

Thank you very much. We have reduced the contents significantly in both results (e.g. – second-order analysis) and discussion sections to avoid repetition. Please see the revised results and discussion sections.

Themes identified are exactly same as topics used to structure the survey. Which demonstrate the limitations of the missing methodological orientation. Why did the researchers not use a more participatory approach and technique in the focus group discussion?

Thank you very much, the themes identified in this qualitative study closely mirror the domains used to structure the focus group guide and the accompanying survey. This overlap reflects the reality that the issues explored—awareness gaps, cultural beliefs, first-aid knowledge, role perception, and systemic barriers—were consistently emphasised by participants as central to their experience. The congruence between survey domains and emergent themes reinforces the validity of these areas as key priorities for PHMs. However, we recognise that the structured nature of the guide may have limited opportunities for participants to raise entirely new topic areas outside the pre-identified domains.

We have updated the limitations section accordingly to acknowledge this fact- “Lastly, the themes identified largely reflected the domains used to structure the focus group guide and survey; while this consistency supports the salience of these issues, it may also reflect the influence of a pre-determined framework, potentially limiting the emergence of entirely new themes.”

Why do the authors think it’s problematic not being able to identify venomous vs non-venomous bites? Thinking about prevention it may be safe to avoid human-snake encounters with all snakes?

Thank you very much for the comment. While it is theoretically safe to advise avoiding all human-snake encounters, in reality, this is often not practically possible due to the environmental context and other threats people face in the setting where the research was conducted. In the reported study setting, people live and work in close proximity to snake habitats. Avoiding all snakes completely can be extremely difficult and sometimes unsafe, as activities such as farming, collecting firewood, or accessing water sources necessitate exposure to natural environments where snakes are present. Moreover, fear of snakes can sometimes lead to unsafe behaviours, such as attempting to kill or handle snakes, which may increase bite risk. The diversity of snake species—both venomous and non-venomous—further complicates the community’s ability to accurately assess risk in the moment.

The findings suggest the researchers think midwives should be responsible for reporting paediatric incidents?

Thank you very much for the comment. While the findings highlight that PHMs are often informally aware of paediatric snakebite incidents in their communities, the study does not advocate assigning them formal responsibility for case reporting. However, their close community contact positions them well to contribute to improved surveillance if supported by clear guidelines, training, and integration into existing health information systems.

We do not know how big the snakebite envenoming problems is compared to the other probably more frequent medical problems the PHMs are working on. How can the researchers justify it’s medical beneficial to take time from midwives dedicated to a less common problem in their communities; which disease/intervention/activity would need to be reduced?

Thank you very much for the comment. We acknowledge that the overall burden of paediatric snakebite envenoming in a given PHM’s catchment area may be lower than that of more frequently encountered conditions such as malnutrition, respiratory infections, or vaccine-preventable diseases. However, snakebite envenoming carries a uniquely high risk of rapid deterioration, long-term disability, and death—particularly in children—if appropriate first aid and timely referral are not initiated. In rural Sri Lanka, delays in care are often driven by preventable factors such as harmful traditional practices, lack of first-aid knowledge, and poor transport links. PHMs, as the first and often only health contact in remote areas, are strategically placed to address these factors during their routine interactions with families, especially in high-incidence districts. We are not suggesting that snakebite prevention displace core maternal and child health activities; rather, it can be integrated into existing health promotion platforms—such as school visits, maternal clinics, and home visits—without significantly increasing workload. By embedding brief, context-specific prevention and first-aid advice into these established activities, PHMs can help avert the severe outcomes of this otherwise low-frequency but high-severity condition.

Why do authors believe a intervention looking at prevention and snakebite response would require an effort focused on children only and another one focused on adults? How likely is it for a neglected tropical disease with limited budget to be able to design such interventions for different target groups instead of an overall prevention intervention in the community?

Thank you very much for this important question. We recognize that, given limited resources typical of neglected tropical disease programs, designing entirely separate interventions for children and adults may not always be feasible. However, children and adults often differ significantly in their risk exposures, behaviours, and physiological vulnerabilities related to snakebite. For example, children’s play activities and smaller body size may increase both risk and severity, while adults face occupational hazards and different prevention challenges. Given these differences in risk, we suggest that brief educational interventions be designed to deliver key messages to rural high-risk communities through existing healthcare delivery systems without overburdening them.

In the abstract the authors conclude the PHMs to represent and ‘underutilized’ workforce. Their findings nevertheless suggest the PHMs to be an ‘overutilized’/ overburdened workforce not able to pick up extra tasks without further investments in the size of the workforce. PHMs indicate to be overburdened and seem to instead suggest to utilize other community platforms to teach communities on prevention and response. There’s a mismatch between the reported statements from the PHMs and the researchers’ conclusions.

Thank you very much, we have entirely rewritten the conclusions. Please see the revised conclusion section.

Researchers suggest midwives to play a role in similar settings across the global snakebite belt but nowhere in the paper do the researchers discuss other published literature on the role of community health workers in snakebite response or in other NTDs (eg midwife roles in other NTDs or community health volunteers in snakebite response). Without this context it’s difficult to follow the researchers’ conclusion that other high risk snakebite settings should switch their community activities from the community health workers to the midwives.

Thank you for the comment. We have rewritten the third paragraph in Discussion section only to highlight the importance of incorporating child-focused educational materials in to existing education programs for snakebite prevention and first aid designed for rural at-risk communities.
