# Supplementary material for: Public Health Midwives’ perspectives on paediatric snakebite prevention and management in rural Sri Lanka: A qualitative study
Source: PLoS Negl Trop Dis. 2026 Feb 11;20(2):e0013976. doi: 10.1371/journal.pntd.0013976 (PMC12893596; doi:10.1371/journal.pntd.0013976)
Supplement: Attachment — Submitted filename: Response_to_reviewers_auresp_2.docx [file pntd.0013976.s004.docx]

**Authors’ response to reviewers’ comments**

Dear editor and the reviewers,

We would like to extend our heartfelt thanks to the reviewers for their time, effort, and invaluable feedback in reviewing this manuscript. Your insightful comments and constructive suggestions have significantly enhanced the quality of this work. We greatly appreciate your expertise and dedication, which have contributed to refining our study. Your support in the review process is deeply appreciated, and we are grateful for your commitment to advancing research in this important area.

Kavinda Dayasiri

Corresponding author

Please see the responses below for specific comments.

Reviewer #1: The Results are very long, and almost no reader of this journal will read them all. Please reduce this and use supplementary material to provide the information. In fact the Results could be much shorter, possibly even the first couple of paragraphs with reference to supplementary material. This is not a qualitative research journal.

We appreciate your suggestion to shorten the Results section and move additional details to supplementary material. However, we would like to respectfully clarify that the first paragraph of the Results provides essential demographic information that is critical for contextualizing the study population. The second paragraph introduces themes that are directly connected with the subsequent results and therefore cannot be omitted or moved without disrupting the logical flow. Additionally, Reviewer #2 requested the inclusion of the thematic map as a figure rather than supplementary material, which we have now incorporated into the main manuscript. We have, however, carefully revised the manuscript to address ALL OTHER comments and improve clarity and readability wherever possible. Thank you very much for your comment and understanding.

Reviewer #4: The supplementary figure on the thematic map added to the manuscript is valuable for the direct visualization of the study's findings. However, the quality of the figure is substandard. I would recommend that the authors prepare a better figure using the freely available softwares and include this figure not as supplementary but as a main figure in the manuscript.

Thank you very much for the comment. Thematic map is now included as Figure 2. Figure 2 legend is added to results section. SI are numbered appropriately.

Reviewer #1: The conclusions are far too long, two long paragraphs. You need to summarise the study into 3-4 sentences, so there are take home easy to understand messages. It also needs to be clinically and epidemiologically applicable.

Thank you very much. We have revised the conclusion as follows.

“Public Health Midwives are well positioned to support paediatric snakebite prevention in rural Sri Lanka but currently face gaps in knowledge, training, and operational resources. Strengthening PHM capacity through brief, paediatric-focused training and integrating snakebite education into routine maternal–child health activities could substantially improve community preparedness. These findings offer practical, scalable strategies for reducing paediatric snakebite morbidity in similar rural settings across the global snakebite belt.”

Reviewer #1: The authors need to have the manuscript read by a native English speaker to improve the grammar.

Thank you very much, we now have the manuscript read a native English speaker.

The introduction still contains the term "snakebite envenoming". This should be either "snakebite", which is when a human is bitten by a snake OR "snake envenoming" in which a snakebite results in envenoming. Please correct this throughout as originally asked.

Thank you very much, we have revised the term as you advised.

The author continue to use the term "endemic", this is not an appropriate term for snakebite in a region such as Asia. There are snakes everywhere, and this should simply say in regions in which the incidence of snakebite is high. This is a very important distinction, because "endemic" means nothing in this context, and high rates of snakebite are due to a high rate of the interaction between snakes and humans - which has nothing to do with whether the snakes are endemic or not. Again, this is a misuse of the English and the authors needs to get advice from an English speaker with expertise in snakebite terminology.

Thank you very much for pointing this out, we have revised the sentence in the Introduction section as per your advice.

“Paediatric snakebites represent a substantial proportion of cases in high-incidence regions, and their clinical impact is significant, often requiring specialised assessment and timely management.”

The authors state that paediatric snake bite ... "with higher risks of severe envenoming, long-term disability, and death compared to adult". I am unaware of any evidence to support this. The reference 3. Abouyanis et al. is a study of only paediatric intensive care patients, and no adult snakebite patients. It simply says that the burden of paediatric snakebite is high in relation to other paediatric conditions. They do not compare it to adults and in fact say that the mortality was low. So please remove this because it is untrue, and not a reason to justify a study in paediatrics.

Thank you very much, we agree with your suggestion. Therefore, we have revised the sentence as mentioned below.

“Paediatric snakebites represent a substantial proportion of cases in high-incidence regions, and their clinical impact is significant, often requiring specialised assessment and timely management.”
